# Distributed Random Beacon for Blockchain Based on Share Recovery Threshold Signature

**DOI:** 10.3390/s22166004

**Published:** 2022-08-11

**Authors:** Yan Zhu, Bingyu Li, Yang Yang, Zhenyang Ding, Haibing Zheng, Guangyu He, Shengjie Hou

**Affiliations:** 1 School of Cyber Science and Technology, Beihang University, Beijing 100191, China; 2 Hangzhou Innovation Institute, Beihang University, Hangzhou 310051, China; 3 Neusoft Corporation, Shenyang 110179, China; 4 Liaoning Blockchain Engineering Technology Research Center, Shenyang 110179, China; 5 National Innovation Institute of Defense Technology, Academy of Military Sciences, Beijing 100071, China

**Keywords:** random beacon, blockchain, bivariate polynomial, threshold signature, share recovery

## Abstract

Random beacons play a crucial role in blockchains. Most random beacons in a blockchain are performed in a distributed approach to secure the generation of random numbers. However, blockchain nodes are in an open environment and are vulnerable to adversary reboot attacks. After such an attack, the number of members involved in a random number generation decreases. The random numbers generated by the system become insecure. To solve this problem while guaranteeing fast recovery of capabilities, we designed a threshold signature scheme based on share recovery. A bivariate polynomial was generated among the participants in the distributed key generation phase. While preserving the threshold signature key share, it can also help participants who lost their shares to recover. The same threshold setting for signing and recovery guarantees the security of the system. The results of our scheme show that we take an acceptable time overhead in distributed key generation and simultaneously enrich the share recovery functionality for the threshold signature-based random number generation scheme.

## 1. Introduction

Distributed randomness has played a crucial role since the birth of blockchain technology. In the classic Nakamoto consensus, the winner of the consensus would be inseparable from the generation of publicly verifiable randomness, which is calculated by a hash function. The verifiability of public randomness allows the verifier to quickly determine the validity of the solution to the proof-of-work puzzle.

As blockchain technology evolves, many excellent protocols are being devised. Distributed randomness generation remains a significant protocol component of these protocols. Blockchain protocols that incorporate distributed random beacons as protocol components mainly include proof-of-stake (PoS) consensus [1], leader [2], and committee selection [3] of a Byzantine fault tolerant (BFT) consensus [4], blockchain sharding [5], anonymous selection [6], etc.

The development of blockchain technology has also contributed to the development of the direction of generating distributed randomness by relying on cryptography primitives. Threshold signature (TSS) [3], verifiable delay function(s) (VDF) [7], publicly verifiable secret sharing (PVSS) [8], verifiable random function (VRF) [9], and homomorphic encryption (HE) [10] are essential instruments for constructing distributed random beacons. The design of distributed random beacons has also become the focus of designing an optimized blockchain protocol [11].

Secure distributed random beacons are expected to consistently generate publicly verifiable, unpredictable, bias-resistant randomness. However, distributed randomness generation participants are in open networks. Participants may be subject to attacks by active adversaries [12]. The active adversary may restart the honest random beacon protocol participants. Then the honest participants under such an attack will lose the ability to participate in the protocol, which in turn compromises the security of the randomness generation. Further, it poses a significant threat to the blockchain system. Malicious adversaries can arbitrarily control the output of random numbers. This attack behavior eventually leads to blockchain systems becoming untrustworthy [13].

The group of participants involved in randomness generation in the blockchain is in the public network. The adaptive adversary can corrupt participants in the group. The adversary can adaptively destroy a limited number of nodes over a while. Active adversaries restart the nodes, causing them to lose stored information and thus the ability to participate in random number generation. Therefore, a method that can help participants regain the ability to generate random numbers while ensuring the security of random number generation needs to be considered in the design of distributed random beacon.

Typically, in a random beacon protocol employing threshold signatures, an active adversary may launch an attack on the participants, causing it to lose its own key share. After suffering such an attack, the participants can no longer participate in the threshold signature generation process [14]. This dramatically reduces the security requirements against the bias-resistance initiated by the adversary on the randomness [3], and the adversary can readily gain profit in the protocol [15]. Active adversaries have the ability to interfere with the generation of random numbers. Ultimately, this attack leads to the generation of random numbers becoming insecure.

In this work, a share recovery threshold signature scheme is proposed for the above adversary attack scenario. After a participant is subjected to an active adversary attack, it can restart to obtain the correct share with the assistance of other honest nodes and, thus, continue to receive the randomness generation capability. Our proposed scheme utilizes the dual homogeneous asymmetric polynomial. One dimension of the polynomial is employed for the threshold signature. Another dimension of the polynomial is used to help participants who suffer from active adversary attacks to recover the lost key share. After a theoretic analysis and experiment evaluation, the presented random beacon scheme can guarantee the properties of bias resistance, unpredictability, and public verifiability of randomness.

The main contributions of this paper are as follows.

1. Secure. After the execution of the key generation, the participants have a signature key share and a binary polynomial to assist other participants in recovering the private key share. A dual homogeneous asymmetric polynomial scheme can prevent the adversary from recovering the secret information below the threshold range. The remaining participants can help the lost share participant to recover the private key share by simply issuing lightweight information.
2. Robust. Robust threshold signature schemes are devised. The share recovery mechanism for the threshold signature can be employed to help participants who have lost their key shares recover them. The perfection of the threshold signature key recovery function effectively prevents active adversary attacks and enhances the availability and bias-resistance of the random beacon.
3. Trustworthiness. The process of key generation is performed in a way. The random number generation process does not have any trusted participants. Our proposed scheme addresses the challenge of the dual homogeneous asymmetric polynomial generation.

The rest of this paper is organized as follows. In Section 2, we describe the related work on the distributed randomness beacon. In Section 3, we present the preliminaries of our protocol. Section 4 describes the system model and an overview of our robust distributed beacon. In Section 5, we describe the robust distributed beacon in detail and analyze its correctness and security. Section 6 presents our prototype implementation and evaluation results. In Section 7, we present our conclusions.

## 2. Related Work

Blum’s two-node coin tossing protocol [16] can be considered the beginning of the distributed random beacon research. Since then, a series of technical approaches to distribute random beacons using different models have been introduced [17,18,19]. This paper focuses on the random number generation solutions adopted in the blockchain. Recovering lost key shares is another important topic of discussion in this paper. Key share recovery has been a hot topic in recent research. We also present the state-of-the-art research advances in share recovery.

### 2.1. Simple Approach

The most straightforward approach to obtaining a random beacon is to rely on a public organization or a single node, such as NIST [20], Oraclize.it (accessed on 20 June 2022), [21] and Random.org (accessed on 20 June 2022) [22]. However, the above-described methods may have the risk of backdoor embedding. Blockchain technology completes the trust establishment between peer-to-peer nodes and should not be powered by random external beacons to provide randomness.

### 2.2. Distributed Randomness Beacon

As mentioned above, distributed random beacons play a crucial role in the secure operations of blockchain protocols. The academic community is filled with research on distributed random beacons. In this section, we summarize the design of distributed beacons in blockchain by classifying them according to cryptography techniques.

- Threshold signature-based randomness beacon.
  The core of the threshold signature [23] is to split the secret private key information into multiple participant scenarios, thus achieving multi-party confirmation. In the normal (t,n) threshold signature scheme, *n* denotes the total number of participants and *t* is the threshold value for obtaining a valid signature. When any *t* (or more than *t*) participants sign the same message, the signature of the community for this message is obtained. However, any less than *t* participants (e.g., t−1) are unable to obtain a valid signature. Eventually, any participant can verify the correctness of the signature using the public key. The unpredictable and unique property of the result of the threshold signature is an excellent random beacon. DFINITY is a typical project in blockchain research that employs threshold signatures as a random source [3]. Participants are randomly assigned to different committee members based on the random number set in the genesis block. A distributed key generation algorithm is run within each committee to generate the private key share of each participant and the verified public key of the committee. The committee members adopt the last round of random numbers as messages and generate a BLS signature [24]. Each participant who collects a valid signature share that satisfies the threshold can recover a unique valid signature. The uniqueness of the threshold signature guarantees that the correct signature recovered by all nodes is the same for all participants. There is no divergence in the final signatures due to the different selected sets of signature shares. The final signatures are treated as inputs of the VRF to obtain randomness for this round.
- Verified random function-based randomness beacon.
  Verified random functions have evolved from the pseudorandom oracle [25]. The pseudorandom oracle enables the input of an initial seed *s* that can map a random sequence of *a* bit-lengths to a pseudorandom sequence of *b* bit-lengths. The output pseudorandom sequence is indistinguishable in polynomial time from the *b* bit-length random sequence. The pseudorandom oracle cannot be employed as a distributed random beacon because the randomness of the random output sequence is not verifiable. Goldreich et al. [26] proposed a verifiable random function to address this issue. For input *x*, the output of the verified random function cannot be computed in polynomial time, and the correctness of the output can be verified. In the blockchain protocol research, Ouroboros Praos [1], Algorand [4], RandChain [27], and DFINITY [3] use this component as part of the protocol. In a recent study, two verified random functions were proposed and analyzed by strict cryptography; the random numbers they output had strong bias-resistance and pseudo randomness properties.
- Verified delay function-based randomness beacon.
  Boneh et al. [7] proposed a technique called the verifiable delay function. During the computation of the function, multiple processors cannot be in parallel to obtain the result faster. A predetermined amount of time must elapse before the calculator obtains the result. Moreover, the result of the computation can be verified relatively rapidly [28]. This feature makes it impossible for the calculator to predict the outcome, so the final output is unpredictable randomness. Later, Lenstra and Wesolowski proposed a slow-time hash function sloth to construct a verified delay function that allowed multi-participant input (outputting a random result). This makes the verified delay function a better distributed random beacon. RANDAO [29] is an Ethereum smart contract based on a verified delay function. Participants submit their local randomness to the smart contract. After calculation, the smart contract outputs global randomness.
- Public verified secret sharing-based randomness beacon.
  Classical secret-sharing schemes share a secret message among a group of participants, with a specified number of authorized users participating to recover it by a specific method. With large-scale applications, the verification of the correctness of the secret share becomes an important issue. Both the shares given by the dealer to the participants and the shares used by the participants for reconstruction can be incorrect, resulting in the secrets not being reconstructed. The proposal of verifiable secret sharing ensures that the correctness of shares can be verified before the dealer and the participant. Feldman’s verifiable secret-sharing [30] scheme provides verifiability, correctness, and privacy. Stadler [31] proposed publicly verifiable secret sharing. Any arbitrary user in the system can verify the correctness of the share by available information. The publicly verifiable feature makes publicly verifiable secret sharing an essential component of distributed random beacon. Distributed random beacon schemes based on verifiable secret-sharing constructs are popularly employed in the blockchain. The following is an example of Ouroboros, which describes the general working process of publicly verifiable secret sharing. However, this orientation is not the primary focus of the article’s research. Randomness generation in Ouroboros consists of two phases: commit and open. In the commit phase, participants encrypt the shared information by running PVSS. The participants submit the communicated information on the blockchain. In the open phase, each participant decrypts all of the encrypted shares using the public key. Then, each participant uses the decrypted shares to compute a local random value, publishing it to the blockchain. Finally, the beacon output is calculated by performing an XOR operation on all published local random values. Recently, Bhat [32] proposed OptRand based on PVSS. OptRand employed PVSS and non-interactive zero-knowledge proofs to build a linear size publicly verifiable random sharing.
- Decentralized randomness from the blockchain.
  The blockchain itself has a lot of randomnesses. The collection of arbitrary transactions in the block and the unpredictability of signatures of transactions are potential sources of randomness [33]. Although there are some applications in lottery gaming, the proof of bias resistance for these protocols is a difficult challenge to cross.

### 2.3. Share Recovery

The study of share recovery has been a key topic of research in the field of secret sharing. The research focuses on two aspects. One is the scenario under active adversary attack, where the adversary reboots the participant, resulting in the loss of key share. The second is in asynchronous verifiable secret sharing. There is an issue pertaining to how to overcome the problem that some participants do not receive the secret share due to the transmission delay.

- Verified secret sharing with share recovery.
  In active adversary attack research, the key consideration of the share recovery scheme is not to give the recoverer the ability to reconstruct the secret. The earliest research traces back to Herzberg [12]. They proposed a scheme in which proactive secret sharing was used. However, the complexity of the scheme was high; the remaining nodes needed to generate a polynomial for the recovery node and the communication complexity was similar to the distributed key generation. A similar scheme was used in MPSS [34] in combination with the PBFT consensus process. The key recovery process was applied to prevent new group members from gaining access to the key while they gained access to key shares. Adversary capability in our research followed Herzberg’s study. In this work, a more efficient scheme was proposed. The remaining participants did not generate independent polynomials for shares. In another class of studies, a single secret sharing could contain multiple secrets by batching the secret sharing [35]. The overhead of average communication complexity was reduced in this way. Recently Basu [36] proposed the use of a distributed pseudorandom function (DPRF) for efficient secret sharing. The DPRF was used as a mask for the original polynomial share, and the key recoverer *i* could remove the value taken by the DPRF at *i*. The share was recovered efficiently by the above approach.
- Asynchronous verified secret sharing.
  The first practical verifiable secret sharing scheme was proposed by Cachin [37]. This scheme uses a binary polynomial S(x,y) where each participant *i* obtains S(i,x) and S(y,i). If a participant loses a share, it can be recovered by f+1 evaluations S(i,x) and f+1 evaluations S(y,i). The recovery process requires $O(n^{2})$ communication overhead. The asynchronous network assumption model in blockchain becomes a priority consideration. The asymmetric bivariate polynomial (k−1,f) was used in the HAVSS [38] scheme proposed by Kogias. Asymmetric bivariate polynomial k−1 dimension is employed as the key recovery. Alhaddad proposed the use of a “two-layer secret sharing” scheme, HAVEN [39]. The trusted dealer constructs a degree *f* polynomial R(x) of secret R(0). Then for each share R(i), a polynomial degree *f* polynomial Q(x) is constructed (Q(0)=R(i)). Although the above asynchronous verifiable secret sharing has a different purpose than our secret recovery scheme, it provided us with a lot of inspiration.

Based on the above study on share recovery, our comparison of the share recovery scheme is shown in Table 1.

## 3. Preliminaries

In this section, we introduce the cryptographic preliminaries used in our scheme.

### 3.1. BLS Signature

The most well-known threshold signature scheme is the pair-based threshold signature scheme [24]. The BLS signature consists of Setup, KeyGen, SigGen, and Verification—four polynomial algorithms.

- Setup. The BLS signature uses bilinear pairing with a non-degenerative property. Gap Diffie–Hellman groups $G_{1},G_{2}$ of suitable elliptic curve points with values in a group of units $G_{T}$. For each group, set the generator $g_{1}\in G_{1},g_{2}\in G_{2},g_{T}\in G_{T}$. The BLS signature also needs a hash function $H_{1}:{\left\{0,1\right\}}^{*}\to G_{1}$ with values in $G_{1}$.
  (1) $$e:G_{1}\times G_{2}\to G_{T}$$
- KeyGen. Generate a secret key and public key based on the parameters chosen in the setup phase.
  Step 1: select a random integer *x* as the secret key in group $G_{1}$.
  Step 2: compute the public key $Y=g_{1}^{x}$.
- SigGen. Sign a message *m* by the key generated in the KeyGen phase.
  Step 1: compute the hash value $H_{1}\left(m\right)$ of the message.
  Step 2: compute the signature of the message using the secret key *x*.
  (2) $$s={H_{1}\left(m\right)}^{x}$$
- Verification. Verify the correctness of the signatures *s* generated in the SigGen phase. Verify: the signature is only valid if the following equation holds.
  (3) $$e\left(g_{1},s\right)=e\left(y,H_{1}\left(m\right)\right)$$
  Proof of Correctness: the above equation can verify the correctness of the signature for the following reason.
  (4) $$e\left(g_{1},s\right)=e\left(g_{1},H_{1}{\left(m\right)}^{x}\right)=e\left(g_{1}^{x},H_{1}\left(m\right)\right)=e\left(y,H_{1}\left(m\right)\right)$$

### 3.2. Threshold BLS Signature

Boldyreva [23] proposed a well-known threshold BLS signature. Our scheme utilizes the same threshold signature scheme as DFINITY.

- Setup. In the threshold BLS signature, the set of *n* participants $\left\{P_{1},\ldots ,P_{n}\right\}$ along with the secret key set $\left(x_{1},\ldots ,x_{n}\right)$ are used as the (t,n)-threshold Shamir secret sharing of the value *s*. In this set, any k≤t values from this key set cannot reveal any information about *x*. There exists the Lagrangian interpolation algorithm, which takes as input any *t*, or more values from this key set can output *x*. This algorithm is expressed as:
  $$(t,n)\;\mathrm{secret}\;\mathrm{sharing}\left\{P_{1},\ldots ,P_{n}\right\}:\left(x_{1},\ldots ,x_{n}\right)\overset{\left(t,n\right)}{⟶}x.$$
  The secret set $\left(x_{1},\ldots ,x_{n}\right)$ corresponds to the public key set $\left(y_{1},\ldots ,y_{n}\right)$. The public key set $\left(y_{1},\ldots ,y_{n}\right)$ and *x* corresponding *y* are available to all participants.
- SigshareGen. Participants $\left\{P_{1},\ldots ,P_{n}\right\}$ sign a message *m* by the key generated in the KeyGen phase.
  Step 1: participants compute the hash value $H_{1}\left(m\right)$ of the message.
  Step 2: participants $\left\{P_{1},\ldots ,P_{n}\right\}$ compute the signature of the message using their own secret key $x_{i}$ where i∈[1,n] :
  (5) $$s_{i}=H_{1}{\left(m\right)}^{x_{i}}$$
  The participant calculates the share of signatures and broadcasts it to the other participants.
- Sigreconstruction.
  Step 1: After obtaining the signature shares of others, participants $\left\{P_{1},\ldots ,P_{n}\right\}$ verify the correctness of $s_{i})$. The verification process uses the same equation as the BLS signature above. The correctness of the equation is the same as the verification of the correctness of the BLS signature. The above signature correctness verification is passed and the signature reconstruction operation is performed.
  Step 2: any t+1 or more correct shares of subset *R* compute the signature:
  (6) $$s=\Pi_{i\in R}\left(s_{i}^{L_{i}}\right)$$
  where $L_{i}$ is the appropriate Lagrange coefficient for the correct signature share set.
- SigVerification. Verify the correctness of the signatures *s* generated in the Sigreconstruction phase. This phase is the same as the BLS signature.
  Verify: the signature is only valid if the following equation holds.
  (7) $$e\left(g_{1},s\right)=e\left(y,H_{1}\left(m\right)\right)$$
  Proof of Correctness: the above equation can verify the correctness of the signature for the following reason.
  (8) $$e\left(g_{1},s\right)=e\left(g_{1},H_{1}{\left(m\right)}^{x}\right)=e\left(g_{1}^{x},H_{1}\left(m\right)\right)=e\left(y,H_{1}\left(m\right)\right)$$

### 3.3. Decentralized Key Generation

To implement a threshold signature key generation system between participants, the distributed key generation phase needs to be applied. In order to implement the recovery of the lost key shares, our scheme improves on the existing basic protocol. Improvements of the distributed key generation are described in detail in Section 5.

- Setup. In the setup phase, some public parameters were created.
  $G_{q}$ is the subgroup of $F_{p}$ of the order *q*, where *p*, *q* are both large primes, *q* divides p−1, and *g* is the generator of $F_{p}$. Our scheme denotes a group of *n* participants as $\left\{P_{1},\ldots ,P_{n}\right\}$.
- KeyGen. The threshold public key *y* is constructed by the share public keys of all members.
  Step 1: $P_{i}$ computes its share public key $y_{i}=g^{x_{i}}$.
  Step 2: $P_{i}$ broadcasts a commitment $C_{i}=C\left(x_{i}\right)$ to all participants.
  Step 3: every participant computes the public key $y=\prod_{i=1}^{n}y_{i}$. *y* can verify the correctness of the reconstructed signature; thus, the threshold secret key $x=\sum_{i=1}^{n}x_{i}$.
- Broadcast. $P_{i}$ shares its own generated polynomial $f_{i}\left(z\right)$ to all of the participants without revealing the coefficients.
  Step 1: construct a random polynomial $f_{i}\left(z\right)\in Z_{q}$ of degree *t*, such that the secret key $x_{i}=f_{i}\left(0\right)$. Let
  (9) $$f_{i}\left(z\right)=f_{i0}+f_{i1}z+\ldots +f_{i,t}z^{t}$$
  where $f_{i0}=x_{i}$.
  Step 2: compute commitment $C_{ij}=g^{f_{ij}}$, where j=0,1,…,t.
  Step 3: broadcast ${\left(C_{ij}\right)}_{j=0,1,\ldots ,t}$ and $s_{ij}=f_{i}\left(j\right)$ to other participants. At this time, $C_{i0}=y_{i}$.
- Verification. $P_{j}$ verifies the correctness of $s_{ij}$ sent from  $P_{i}$.
  Step 1: $P_{i}$ computes $s_{ij}=f_{i}\left(j\right)=f_{i0}+f_{i1}j+\ldots +f_{i,n}j^{t}$.
  Step 2: $P_{i}$ sends $x_{ij}$ with the corresponding signature to $P_{j}$ through a secure channel
  Step 3: $P_{j}$ verifies the signature and then checks the correctness by the following equation.
  (10) $$g^{x_{ij}}=\prod_{m=0}^{t+1}{\left(C_{il}\right)}^{j^{m}}$$
  If the condition is not satisfied, the interaction will end. The subsequent interaction in the above case is an interesting issue, but not the focus of this paper. Moreover, $P_{j}$ will broadcast the error to all members.
- Reconstruction. By defining $f\left(z\right)=f_{1}\left(z\right)+f_{2}\left(z\right)+\ldots +f_{n}\left(z\right)$, $P_{i}$ could compute $s_{i}=\sum_{j=1}^{n}s_{ij}=f\left(i\right)$. Thus $s_{i}$ is a share of f(0)=x.
  $P_{i}$ computes its share $s_{i}=\sum_{j=1}^{n}s_{ji}$ where $s_{ji}$ is received from other *j* participants and its own $s_{ii}$. Afterward, $s_{i}$ can be used as the private key share of the threshold signature.

### 3.4. Verifiable Random Function

The verifiable random function is the final progress of our scheme to generate randomness. A verifiable random function is defined as a tuple of the following algorithms.

- KeyGen. Input value *r*; the algorithm generates a secret key sk and an output verification key vk.
- Eval. The evaluation algorithm produces a pseudorandom output *R*, the output corresponding proof π on input sk, and a message *m*.
- Verify. Verify the algorithm outputs 1 if and only if the output produced by the evaluation algorithm is *R* and is verified by the proof π given the verification key vk and the message *m*.

## 4. Decentralized Random Beacon Committee for Blockchain

In this section, an overview of the random beacon committee for blockchain is presented, including the application scenario, system architecture, and security properties.

### 4.1. Application Scenario

Random beacon committees can be very instrumental for blockchain. DFINITY uses this approach to assign different participants to different committees. However, members of the committee may be attacked by active adversaries. Active adversaries launch reboot attacks against some of these members. Members lose their secret share, which in turn leads to the loss of the ability to participate in randomness generation. This eventually results in the random number output of the committee becoming insecure. The adversary can easily influence the entire committee. Ultimately, the above attack behavior leads to the generation of biased randomness.

Addressing this issue allows for capacity enhancements to the existing blockchain. On the one hand, the key recovery process requires a straightforward process. Share recovery for any one participant does not cause all nodes to change. The scenario for share recovery should be provisioned during protocol initialization. On the other hand, the components of the system cannot have a trusted third party. Therefore, the design of our protocol focuses on the design of polynomials that initialize the decentralized key generation.

### 4.2. System Architecture

**Figure 1:**
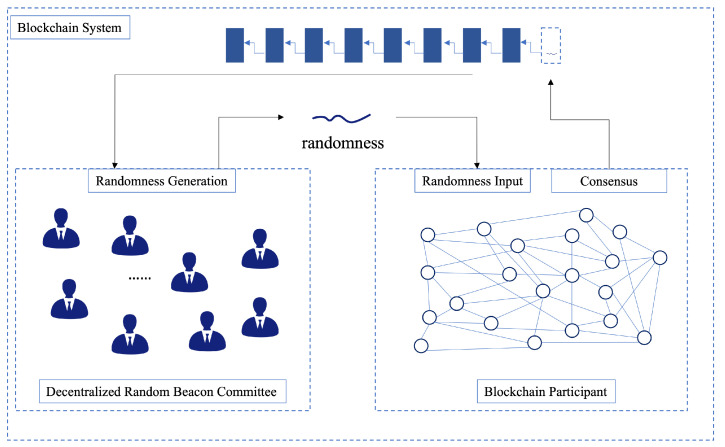
The architecture of our scheme.

The randomness generation beacon committee for blockchain consists of three sub-modules: blockchain participant, decentralized random beacon committee, and blockchain system. The system architecture is depicted in Figure 1.

- Blockchain participants are the base members of the blockchain. They are composed of different committees for normal transaction validations according to the randomnesses generated by the random number committee. The participants perform the basic processes of transaction initiations, confirmation, and consensus of the blockchain.
- The decentralized random beacon committee is the core of the system. Committee members run the distributed key generation. After the key generation, the committee members run the distributed threshold signature scheme and output the threshold signature shares. After the signature share reconstruction process, the final signature is an output. It is worth noting that the reconstructed signatures are verified for correctness by the public key and then a consistent threshold signature is an output. The final threshold signature is input to the verifiable delay function to output the final random number. The random number obtained by the committee is used to determine the committee composition of the participants for the next round.
- The blockchain system records the transactions in which the nodes operate normally. Meanwhile, the randomness generated in a round is recorded in each block in order to implement the next round of randomness generation. This random number is used in the next round of signed messages to generate randomnesses.

### 4.3. Security Properties

The randomness generation beacon committee for blockchain that we proposed was designed to achieve the following property requirements. For the unpredictability, bias-resistance, public verifiability, and availability properties, we refer to the excellent work by Raikwar [11]. The following mathematical formulas are referenced from the work by Raikwar. The recovery property was due to the new security requirements brought about by our solution design. In the following, definition λ is a security parameter and negl(λ) is a negligible function of λ.

- Unpredictability. Let $A\left\{s_{1},\ldots ,s_{e}\right\},st_{e})$ be a probabilistic polynomial time algorithm that receives secret shares $s_{1},\ldots ,s_{e}$ where (e≤t) and the current state $state_{e}$ as the input values. Let A output the a value $s_{e+f}$ for any value (future rounds) f≥2, and for all rounds e≥1. The following relation is satisfied.
  (11) $$\mathrm{Pr}\left[A\left(s_{1},\ldots ,s_{e},state_{e}\right)=s_{e+f}\right]\leq \mathrm{negl}\left(\lambda \right)$$
- Bias resistance. Let $A_{i}\left(s_{1},\ldots ,s_{e-1}\right.$, $state_{e-1}$) for i=1,…,e where (e≤t) be probabilistic polynomial time algorithms that receive the values $v_{1},\ldots ,v_{e-1}$ and the current $state_{e-1}$ as input and output one bit: 0 or 1. Let bit ${}_{i}\left(s_{e}\right)$ denote the *i*-th bit in the binary representation of $s_{e}$, let $b=\left|s_{e}\right|$ be the number of bits of $v_{e}$. Then, for every e≥1, every $A_{i}\left(\right)$, and for all (i=1,…,e).
  (12) $$\mathrm{Pr}\left[{\mathrm{bit}}_{i}\left(s_{e}\right)=A_{i}\left(s_{1},\ldots ,s_{e-1},state_{e-1}\right)\right]\leq \frac{1}{2}+\mathrm{negl}\left(\lambda \right)$$
- Public verifiability. Verify( ) as a public probabilistic polynomial time algorithm run by an external randomness verifier. The verifier at the end of round *e* receives $v_{e},\pi_{e}$ and the $state_{e-1}$ as input values, and outputs a bit 0 or 1 based on the verification of $v_{e}$ using $\pi_{e}$. Then, for every round e≥1.
  (13) $$\mathrm{Pr}\left[\mathrm{Verify}\left(v_{e},\pi_{e},state_{e-1}\right)\neq 1\right]\leq \mathrm{negl}\left(\lambda \right)$$
- Availability. Let A be an adversary controlling a fraction of participants and $P^{h}\subseteq P$ be a set of honest participants in the decentralized randomness beacon protocol. The number of $P^{h}$ is more than t+1. Given $v_{e},\pi_{e},params$ and $state_{e-1}$, for every round e≥1 and for every participant $P_{i}\in P^{h}$.
  (14) $$\mathrm{Pr}\left[\;\mathrm{UpdateState}\;\left(state_{e-1},params,v_{e},\pi_{e}\right)\neq state_{e}\right]\leq \mathrm{negl}\left(\lambda \right)$$
- Recovery. Let A be an adaptive adversary rebooting a fraction of participants and $P^{d}\subseteq P$ be a set of dishonest participants in the decentralized randomness beacon protocol. The number of $P^{d}$ is less than *t*. Given $v_{e},\pi_{e},params,$ and $state_{e-1}$, for every round e≥1 and for every participant $P_{i}\in P^{h}$.
  (15) $$\mathrm{Pr}\left[\;\mathrm{UpdateState}\;\left(st_{e-1},params,v_{e},\pi_{e}\right)\neq state_{e}\right]\leq \mathrm{negl}\left(\lambda \right)$$

## 5. Decentralized Random Beacon with Share Recovery Threshold Signature

In this section, we present a process description of the decentralized random beacon with the share recovery threshold signature, including the system definition and construction. Then we give the correct analysis and security analysis of the proposed scheme.

### 5.1. System Definition

A share recovery threshold signature mainly consists of the following six algorithms: setup, distributed key generation, share recovery, threshold signature, signature verification, and randomness generation.

- Setup. This step runs to initialize the scheme. It takes as input a security parameter $1^{k}$, and outputs the system public parameter params.
- Decentralized key generation. Decentralized random beacon committee members $\left\{P_{1},\ldots ,P_{n}\right\}$ take public parameters params as input and run this step to generate their own key share $\left\{Msk_{i}\left(i=1,\cdots ,n\right)\right\}$, recovery polynomial $R_{i}\left(i,y\right)$, and public signature verification key Cpk.
- Share recovery. Once a participant $p_{i}$ of the committee loses his share of the key, the rest of the participants assist him in recovering the key share. Other participants send $R_{i}\left(x,i\right)$ to $p_{i}$. After receiving more than t+1 recovery shares, he can recover the signature shares $Msk_{i}$ himself.
- Threshold signature. $\left\{P_{1},\ldots ,P_{n}\right\}$ participants take as the input the system public parameters params and the message recorded in the last round block *m*; they share their own key share $\left(Msk_{i},i=1,\cdots ,n\right)$ and output threshold signature σ.
- Signature verification. Committee members $\left\{P_{1},\ldots ,P_{n}\right\}$ verify the validity of the signature σ. It takes as the input the system public parameters params, message *m*, signature σ, the shared signature verify public key Cpk, and the output 1 if and only if the unique signature is valid (otherwise outputs 0).
- Randomness generation. The unique verified signature σ is entered into the verifiable random function for the calculation. The output of the randomness calculation and the evidence $\pi_{e}$ of the calculation are stored in the block of the current round.

### 5.2. Random Beacon with Share Recovery Threshold Signature Construction

- Setup. This involves the gap Diffie–Hellman groups $G_{1},G_{2}$ of suitable elliptic curve points with values in a group of units $G_{T}$. For each group, we set the generator, $g_{1}\in G_{1},g_{2}\in G_{2},g_{T}\in G_{T}$. Their relationship *e* satisfies Equation (3). A one-way hash function $H_{1}:{\left\{0,1\right\}}^{*}\to G_{1}$ with values in $G_{1}$. *E* is an elliptic curve over $F_{q}$. *g* is a generator on the curve *E* and its order is prime *q*. We also need the same as DFINITY [5] to calculate the obtained threshold signature eventually to the VRF. So, we need the committee’s VRF private key $vrf_{sk}$ and verification public key $vrf_{pk}$. The system parameter is $params=(F_{q},E,g,q,g_{1},g_{2},H_{1},e,vrf_{sk},vrf_{pk})$.
- Decentralized key generation. All committee participants $\left\{P_{1},\ldots ,P_{n}\right\}$ generate the threshold signature key via the distributed key generation, Algorithm 1. It is important to emphasize that the polynomials we use are not symmetric bivariate polynomials $\left(F_{i}\left(a,b\right)\neq F_{i}\left(b,a\right)\right)$. Moreover, the bivariate polynomial dimension has the same degree *t*. Unlike the previous work, we refer to this as the homogeneous bivariate polynomial. The participants interact with each other by the described algorithm. Eventually, they complete the interaction, participant $p_{i}$ will obtain a commitment Commit about the polynomial $F\left(x,y\right)=\sum_{i=0}^{n}F_{i}\left(x,y\right)$, recovery polynomial $R_{i}=\sum_{i=0}^{n}F_{i}\left(x,i\right)$. $p_{i}$ also have $Msk_{i}=\sum_{i=0}^{n}F_{i}\left(i,0\right)$, and the verification public key $Cpk=g^{\sum_{i=0}^{n}F_{i}\left(0,0\right)}$ for the threshold signature. Ultimately, the secret private key for the threshold signature is $Msk=\sum_{i=0}^{n}F_{i}\left(0,0\right)$. It can be the Lagrange reconstruction by the $\left\{Msk_{1},\ldots ,Msk_{i}\right\}$ algorithm. They both satisfies the F(x,0) polynomial distribution. The following mathematical expressions were designed by the authors.

**Algorithm 1** Decentralized key generation for the participant $p_{i}$1:**upon** setup finished **do**2:choose a random homogeneous bivariate polynomial $F_{i}\left(x,y\right)$ of degree (t,t) with $F_{i}\left(0,0\right)=sk_{i}$, i.e.,

$$F_{i}\left(x,y\right)=\sum_{m,n=0}^{t,t}u_{mn}x^{m}y^{n}$$

3:   $Commit_{i}=C_{mn}=g^{u_{mn}}$ for m∈[0,t] and n∈[0,t]▹ $Commit_{i}$ is a matrix4:**set** $Commit=Commit_{i}$5:

$Msk_{i}=F_{i}\left(i,0\right)$

6:

$R_{i}\left(x\right)=F_{i}\left(x,i\right)$

▹ $R_{i}\left(x\right)$ is a polynomial of $F_{i}\left(x,y\right)$ where y=j7:

$Cpk=g^{F_{i}\left(0,0\right)}$

8:9:**for** j∈[1,n] **do**10:

$\;a_{j}\left(x\right)\leftarrow F_{i}\left(x,j\right)$

11:

$\;b_{j}\left(x\right)=F_{i}\left(j,0\right)$

▹ $b_{j}\left(x\right)$ is a value12:**send** “send, $Commit_{i},a_{j}\left(x\right),b_{j}\left(x\right)$” to $p_{j}$13:**upon receiving** $“send,Commit_{j},a_{i}\left(x\right),b_{i}\left(x\right)”$ from $p_{j}$ **do**▹ $p_{i}$ do14:
**check the correctness of $a_{j}\left(x\right),b_{j}\left(x\right)$ by $Commit_{j}$**
15:
**upon correct**
16:
**$p_{i}$ set**
17:

$\;Commit=Commit_{j}\circ Commit$

▹ ∘ is Hadamard product18:

$\;R_{i}=R_{i}+a_{j}\left(x\right),$

19:

$\;Msk_{i}=Msk_{i}\times b_{j}\left(x\right)$

20:

$\;Cpk=Cpk\times g^{F_{j}\left(0,0\right)}$

▹ $g^{F_{j}\left(0,0\right)}$ is extracted from $Commit_{j}$21:**return** $Commit,Msk_{i},R_{i},Cpk$

**Figure 2:**
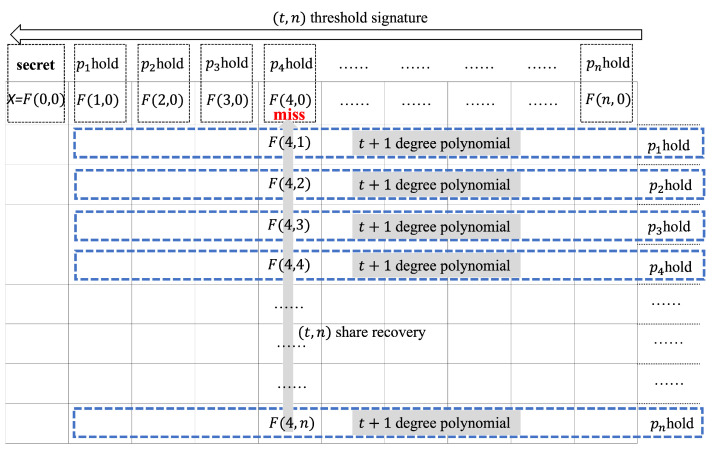
The progress of our share recovery.

- Share recovery. Our scheme assumes that the active adversary launches a reboot attack on no more than n−(t+1) participants (at least t+1 honest participant alive). The process of key recovery is illustrated in Figure 2. The attacked participant $p_{i}$ can recover the key share via Algorithm 2. The following mathematical equations were performed by the authors.

**Algorithm 2** Share recovery for participant $p_{i}$1:**upon** reboot attack effect **do**2:**send** “help, $p_{i}$” to $p_{j}$3:**upon receiving** “help, $p_{i}$” from $p_{i}$4:$p_{j}$ **do** $R_{j}\left(i\right)=F\left(i,j\right)$5:**send** “echo, $Commit,R_{j}\left(i\right)$, $p_{i}$” to $p_{i}$6:**upon receiving** “echo, $Commit,R_{j}\left(i\right)$, $p_{i}$” from $p_{j}$7:
**check the correctness of $R_{j}\left(i\right)$ by Commit**
8:
**upon correct**
9:

$Recovery_{i}\leftarrow Recovery_{i}\cup R_{j}\left(i\right)$

10:**if** echo ≥ t+1 **then** Lagrange from $Recovery_{i}$▹ satisfy F(i,y) polynomial distribution11:
**return**

$Msk_{i},Cpk$

▹ Cpk is extracted from Commit

- Threshold signature. Participants $\left\{P_{1},\ldots ,P_{n}\right\}$ sign a message from the last round block *m* by the decentralized key generation. Participants compute the message hash $H_{1}\left(m\right)$. Then, the participants $\left\{P_{1},\ldots ,P_{n}\right\}$ compute the signature of the message using their own secret key $Msk_{i}$ where i∈[1,n] :
  (16) $$\sigma_{i}=Msk_{i}H_{1}\left(m\right)$$
  The participant calculates the share of signatures and broadcasts it to other participants. After obtaining the signature shares of others, the participants $\left\{P_{1},\ldots ,P_{n}\right\}$ verify the correctness of $\sigma_{i}$. The above signature correctness verification is passed and signature reconstruction is performed. Any t+1 or more correct shares subset *R* compute the signature:
  (17) $$\sigma =\Pi_{i\in R}\left(\sigma_{i}^{L_{i}}\right)$$
  where $L_{i}$ is the appropriate Lagrange coefficient for the correct signature share set.
- Signature verification. Verify the correctness of the signatures σ generated in the reconstruction phase. This phase is the same as the BLS signature. The signature is only valid if the following equation holds.
  (18) $$e\left(g_{1},\sigma \right)=e\left(Cpk,H_{1}\left(m\right)\right)$$
- Randomness generation. After the threshold signature and signature verification phase, the committee obtains a uniquely determined threshold signature. The threshold signature can be input to a verifiable random function for the calculation. The decentralized random beacon committee inputs signature σ with $vrf_{sk}$ into a verifiable random function. The VRF evaluation algorithm produces a pseudorandom output randomness *R* and the output corresponding proof π on input $vrf_{sk}$ and a message σ. Decentralized random beacon committee participants can verify the algorithm to verify output correctness. It is verified by the proof π, given the verification key $vrf_{pk}$ and the message σ.

### 5.3. Correctness Analysis

The correctness of this decentralized random beacon with the share recovery threshold signature includes four aspects: valid decentralized key generation, share recovery, valid signature, and availability randomness. They respectively depend on:

(1) The generation of key shares $\left\{Msk_{1},\ldots ,Msk_{n}\right\}$ can obtain a valid signature
(2) Participants’ $P_{i}$ key share $Msk_{i}$ can be recovered by $\left\{P_{1},\ldots ,P_{n}\right\}$ where n≥t+1.
(3) The generation of the valid BLS signature σ that could be verified.
(4) The randomness *R* generated by the BLS signature σ and VRF committee secret key $vrf_{sk}$ can be verified.

Specifically, the correctness of our scheme is indicated by the following branches.

- Decentralized key generation correctness.
  According to the decentralized key generation phase, key shares $Msk_{i}=\sum_{j=0}^{n}F_{j}\left(i,0\right)$. The participants interact with each other $\sum_{j=0}^{n}F_{j}\left(i,0\right)=F\left(x,0\right)$. Thus, $\left\{Msk_{1},\ldots ,Msk_{n}\right\}$ satisfy the F(x,0) polynomial distribution. In all, the decentralized key generation is correct.
- Share recovery correctness.
  In the decentralized key generation phase, every participant $P_{i}$ holds a recovery polynomial $R_{i}=\sum_{j=0}^{n}F_{j}\left(x,i\right)$. The participants interact with each other $\sum_{j=0}^{n}F_{j}\left(x,i\right)=F\left(x,j\right)$. During the key recovery process, $P_{i}$ sends F(i,j) to $P_{j}$. The degrees of F(x,y), two dimensions, are both *t*. $\left\{F(i,1)\ldots ,F(i,n)\right\}$ satisfy the F(i,y) polynomial distribution. Therefore, once recovery shares are received that satisfy the threshold t+1, the participant $P_{i}$ can recover the share F(i,0). Thus, the share recovery phase is correct.
- Signature correctness.
  Based on decentralized key generation and share recovery correctness, the committee participants’ secret key $\left\{Msk_{1},\ldots ,Msk_{n}\right\}$ satisfy the F(x,0) polynomial distribution. The threshold signature secret is $sk=\sum_{j=0}^{n}F_{j}\left(0,0\right)=sk_{j}=F\left(0,0\right)$. Moreover, $Cpk=\prod_{i=0}^{n}g^{F_{i}\left(0,0\right)}=g^{F(0,0)}=g^{sk}$. Thus, the share signature can be verified.
- Availability randomness correctness.
  Based on the threshold signature correctness, the threshold signature phase can output a unique signature σ. The reason for the uniqueness of BLS signatures includes two aspects. The first reason is the non-adaptive “random *k* value“ involved in the calculation. Moreover, as in Equation (7), the unique public key is involved in the signature verification. The randomness of the output comes from the one-way function of the signed message *m*.

### 5.4. Security Analysis

As mentioned above, our solution needs to satisfy properties, such as unpredictability, bias-resistance, public verifiability, availability, and recovery. In our scheme, signature unforgeability and share privacy are key to gaining the above properties. These overlapping security properties can be formally reduced to two core security definitions: signature unforgeability and share privacy.

**Theorem** **1.** *The proposed share recovery threshold signature construction is unforgeable under the assumption that the GDH is hard.*

**Proof of Theorem 1.**  The signature in the proposed share recovery threshold signature is based on the threshold BLS signature. Note that the security of the threshold BLS signature scheme has been formally proven under the assumption of GDH in the random oracle model [23,24]. Thus, the proposed threshold BLS signature construction also enjoys unforgeability in the random oracle model under the GDH assumption. □

**Theorem** **2.** *The share in the proposed share recovery threshold signature construction is privacy.*

**Proof of Theorem 2.**  We focus first on the information available to adversary A during the decentralized generation stage. Other participants send A the share generation message $\left(Commit_{j},F_{i}\left(x,A\right),F_{i}\left(A,0\right)\right)$ on each share polynomial $F_{i}\left(x,y\right)$. Because of the DLP hardness assumption, the secret-sharing hiding property guarantees that this is insufficient to distinguish any other point on $F_{i}\left(x,y\right)$ from random with non-negligible probability.

Next, we consider the information available to adversary A during the share recovery stage. If adversary A lost his share and requested recovery, he can only gain more than f+1 points of $F_{i}\left(A,y\right)$. He learns nothing about the secret unless he can distinguish secret $F_{(}i,0)$ from random.

Next, we provide insight into the reconstruction stage. In this stage, other dishonest participants may send him *t* share reconstruction messages. He learns nothing about the secret unless he can distinguish the secret; there is one more share reconstruction message from random.

Finally, we need to consider share privacy during share recovery. Adversary A cannot recover shares of other participants through the key recovery mechanism. Our bivariate polynomials use the same threshold t+1. More importantly, our polynomial is not symmetric ($\left(F_{i}\left(a,b\right)\neq F_{i}\left(b,a\right)\right)$). *t* adversary A cannot recover the secret or recover the secret share of other participants. □

## 6. Evaluation

We compared the schemes based on the above study of the distributed random beacon. Table 2 compares the above scheme and our scheme in terms of setup assumptions, communication overhead, active adversaries, and recovery. The scheme proposed in this paper strengthens the DFINITY scheme against active adversary attacks. Participates in RandRunner and the POW random beacon scheme can compete against active adversaries. However, both schemes require a common reference string as the setup assumption. In our proposed scheme, the initialization of the system is accomplished through the distributed key generation. Our scheme has better performance in terms of trustworthiness.

HAVEN is different from our proposed scenario in terms of the scenarios. It is more concerned with the impact of network assumptions on the scheme. We analyzed our scheme in a theory comparison with HAVEN [39], as shown in Table 3. *E* represents the exponentiation calculation and LO represents the Lagrange interpolation. We have the same computational overhead for the signature generation and share recovery compared to HAVEN. However, our proposed scheme has less computational overhead in the generation phase.

**Figure 3:**
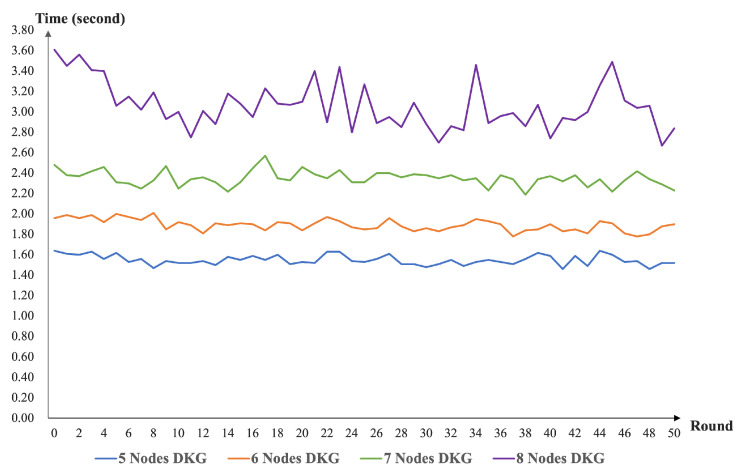
The performance of our DKG scheme with different nodes.

**Figure 4:**
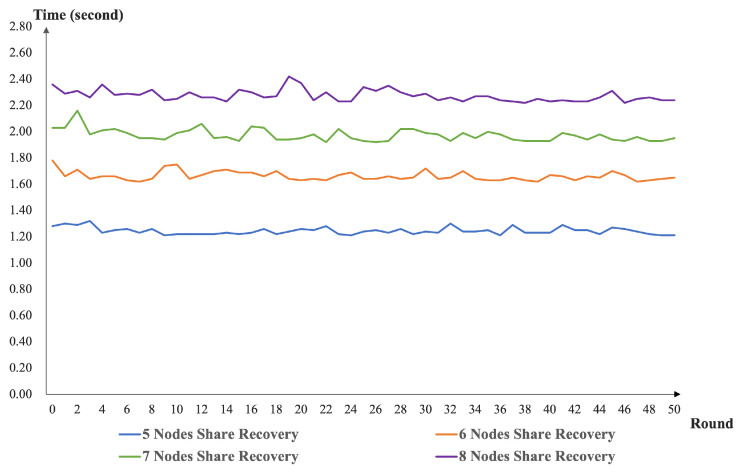
The performance of our share recovery scheme with different nodes.

In terms of the experimental simulation, we implemented the scheme simulation based on the PROJECT [40]. The environment of our simulation was Intel(R) Core(TM) i5-1135G7 @ 2.40 GHz, RAM 16.0 GB, and Ubuntu 9.4.0, JAVA openjdk version 11.0.15. We deployed five to eight nodes to implement the distributed key generation and key recovery. In system deployments with varying node sizes, we executed our program (50 rounds, consecutively) and recorded the execution times. The performances of our DKG scheme and share recovery scheme with different nodes are present in Figure 3 and Figure 4.

**Figure 5:**
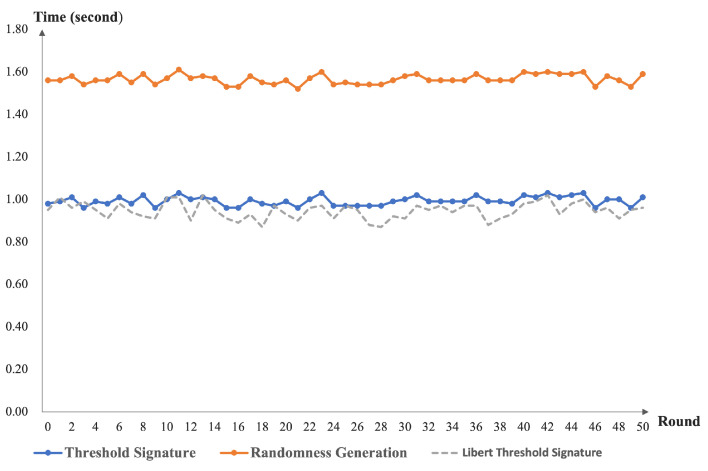
The performance of our threshold signature and randomness generation.

After the DKG execution, we performed the threshold signature and input the results into VRF. Using five nodes as an example, we compared our threshold signature scheme with the Libert threshold signature scheme [41]. Finally, the program output randomness, see Figure 5. It should be noted that the message transmission delay between nodes was considered in our time calculation.

### Performance Analysis

From Figure 3, we can see that our DKG runtime function increased as the number of nodes increased. This is because our scheme is designed to use the homogeneous bivariate polynomial. As the number of nodes increases, the number of interactions between nodes also needs to increase. At the same time, the computation and verification times of the nodes for messages need to increase. This again confirms the design of our solution. As presented in Figure 4, the node share recovery time in our scheme increases as the number of nodes increases. The reason is that the threshold setting for the share recovery is set at the same level as the key recovery setting. From Figure 5, one can see that our scheme has no impact on the efficiency of the threshold signature and VRF computation. The share recovery function was performed before signing. We must enrich the random number generation function based on the threshold signature.

## 7. Conclusions

We presented a key recovery threshold signature randomness beacon scheme for blockchain. This scheme allows participants to recover the key share after an active adversary reboot attack. Moreover, it is proven that our random beacon scheme can avoid generating unpredictability, bias-resistance, and public verifiability randomness. Moreover, our scheme supports the availability and recoverability of randomness generation. As shown in the performance analysis, our practical solution gains new functionality at a fraction of the cost. The number of nodes increases by one node, and the time of DKG and the share recovery add approximately 0.4 s. In the future, additional research needs to focus on the study of the effect of the network assumption on random number generation. At the same time, robust and secure generation of random numbers in asynchronous networks should be investigated. To summarize, due to the recovery and efficiency, our key recovery threshold signature randomness beacon scheme applies to randomness generating for blockchain.

## Figures and Tables

**Table 1:** Comparison of the existing share recovery protocol.

Scheme	Polynomial	Comm. Cost	Trust Setup
Cachine	Bivariate symmetric polynomial	$O(n^{2})$	✗
HAVEN	Two-layer polynomial	O(n)	✗
HAVSS	Asymmetric bivariate polynomial	O(n)	✗
Basu	Polynomial+DPRF	O(n)	✓
Our scheme	Homogeneous bivariate polynomial	O(n)	✗

**Table 2:** Comparison of the existing random beacon protocols.

	Technique	Setup Assumption	Comm. Cost	Adaptive Adversary	Recovery
TSS	DFINITY [5]	DKG	$O(\lambda n^{2})$	✗	✗
TSS	Cachine [17]	DKG	$O(\lambda n^{2})$	✗	✗
VRF	Algorand [4]	CRS	O(λn)	✗	✗
VDF	RandRunner [28]	CRS	$O(\lambda n^{2})$	✓	✓
VSS	Ouroboros [1]	CRS	$O(\lambda n^{4})$	✗	✗
Hash	POW	CRS	O(λn)	✓	✓
TSS	Our scheme	DKG	$O(\lambda n^{2})$	✓	✓

**Table 3:** Comparison of recent asynchronous verifiable secret sharing protocols.

	HAVEN [39]	Our Scheme
Key Generation	$(n^{2}+n)E$	(3n+2)E
Threshold Signature	(p+1)E+LO	(p+1)E+LO
Share Recovery	(p+1)E+LO	(p+1)E+LO

## Data Availability

Not applicable.
